# Mineral and Organic Materials as Factors Reducing the Effect of Petrol on Heavy Metal Content in Soil

**DOI:** 10.3390/ma17143528

**Published:** 2024-07-16

**Authors:** Mirosław Wyszkowski, Natalia Kordala

**Affiliations:** Department of Agricultural and Environmental Chemistry, University of Warmia and Mazury in Olsztyn, Łódzki 4 Sq., 10-727 Olsztyn, Poland; natalia.kordala@uwm.edu.pl

**Keywords:** petrol contamination, soil, materials, heavy metals

## Abstract

As industrial production increases worldwide, so does the demand for fuels. The transport of fuels from the point of production to the end user poses a risk of environmental pollution, both during transport and during combustion in internal combustion engines. The soil is a part of the environment which is particularly sensitive to contamination by petroleum substances. For this reason, research has been carried out into the possibility of reducing the impact of petrol on the content of heavy metals in the soil using various materials, both mineral (bentonite, calcium oxide) and organic (compost). These played an important role in the in situ remediation of contaminated soils. Petrol contamination increased the content of some heavy metals (Pb, Cd, or Ni), while it decreased the content of other metals (Cr, Zn, Co, and Cu) in the soil. The materials used in this study significantly altered the levels of heavy metals in the soil. The strength of the effect varied and the direction of the effect depended on the element. Bentonite was the most effective, while calcium oxide and especially compost were less effective. The most beneficial (limiting) effect of calcium oxide was shown on the soil content of cadmium, cobalt, and chromium, while the bentonite effects were on the content of chromium. The application of the abovementioned materials seems to be effective in reducing low level soil contamination by petrol.

## 1. Introduction

The progress of civilisation and human industrial activities have a negative impact on the natural environment, often leading to its degradation. Among the anthropogenic transformations of soils, their contamination with petroleum substances is of particular importance [1]. This type of xenobiotic alters a soil’s biological properties, affecting its microbial diversity and enzymatic activity [2,3], reduces oxygen and water infiltration [4], alters nutrient content (mainly sodium, potassium, sulphate, phosphate, and nitrate) [5], and reduces soil fertility [6], resulting in stunted growth and reduced crop yields [5,7]. Wyszkowska and Kucharski [8] showed that soil contamination with petrol (6 cm^3^ kg^−1^) reduced the yield of triticale by almost 10 times compared to the control (uncontaminated) group, while also leading to a decrease in soil dehydrogenase and urease activity. In another experiment by Borowik et al. [9], the aboveground biomass yield of maize decreased by 89% and the root yield by 84% after diesel application at a dose of 24 cm^3^ kg^−1^. In addition, soil contamination with crude oil and its derivatives reduces humic and fulvic acids, alters soil redox properties, increases the C:N ratio, reduces nitrification and ammonification [6], and leads to the secondary contamination of groundwater and surface water [10]. 

According to the European Environment Agency (EEA), petroleum hydrocarbons are responsible for 50–60% of soil contamination [11], with the main sources being storage tank leaks, pipeline failures, and the production of refined products [12]. In addition, the illegal or unauthorised extraction of crude oil from pipelines is becoming an increasingly common practice [11], posing a further threat to environmental safety. The presence of hydrocarbons in soils negatively affects the cycling of organic matter and the regulation of active nutrient stocks in soils, as well as soil porosity and texture, thereby storing and moving water and gases and facilitating root penetration [13,14].

Due to their hydrophobic nature and resistance to microbial degradation, petroleum substances have a long half-life in the environment and tend to accumulate in the soil, which can lead to their transfer downstream in the trophic chain [4]. In addition, the polycyclic aromatic hydrocarbons present in petroleum derivatives exhibit mutagenic, carcinogenic, and embryotoxic effects [14], posing serious health risks in industrialised areas and those areas associated with the logistics of petroleum products.

Soil contamination with petroleum derivatives also results in increased levels of trace elements. Excessive amounts of these can have a directly negative effect on crops, the physiological activity of soil microorganisms, and the biochemical activity of the soil [15]. An experiment conducted by Adebiyi et al. [16] showed that the soil adjacent to the Ejigbo Oil Depot terminal in south–west Nigeria had elevated Cu, Ni, and Cr contents and high bioavailability and mobility potentials for Cd and Zn compared to the control soil. A similar trend was reported by Adebiyi and Ayeni [17], who found elevated levels of Pb, Zn, Cd, and Cr and lower levels of Ni, Co, and Mn in soil collected from the site of an oil depot marketing company compared to the control site. Above-optimal levels of trace elements in soil can be toxic to plants, leading to reduced crop productivity as a result of reduced root and shoot growth [18], inhibition of photosynthesis and cellular respiration [19], uptake of water and other nutrients [20], and nitrogen metabolism [21]. The effect of trace element accumulation in plant cells also results in an increased production of the reactive oxygen species (H_2_O_2_, O^−2^, OH^−^), which can induce oxidative stress in cells [22]. Under these conditions, protein and lipid oxidation, cell membrane denaturation, ion release, and DNA damage occur, ultimately leading to the activation of programmed cell death [23]. 

Soils contaminated with petroleum derivatives are excluded from agricultural and recreational uses, and the process of their self-regeneration can take many years, in some cases even decades [6]. Therefore, any attempts to rehabilitate such soils are extremely important and desirable. One of the techniques used to reduce the negative impact of petroleum substances on soil is in situ stabilisation. This method is characterised by its simplicity of implementation and low financial outlay [24]. It also does not destroy the soil’s biological activity or alter the soil’s organic matter content [25]. Various soil amendments are used, including calcium oxide [26,27], biocarbon [28,29], compost [30,31], minerals [32,33,34], ash [35,36,37], or sewage sludge [38,39]. The main function of the aforementioned materials is to reduce the solubility of contaminants and limit their vertical migration and toxicity through the combined mechanisms of adsorption, complexation and precipitation [40,41].

The in situ stabilisation of contaminated soils increases the soil’s sorption capacity, soil pH and organic matter content, influences the redox potential [42,43] and improves the habitat for soil microorganisms that take up and process hydrocarbon contaminants [44]. The above changes lead to a reduction in the contaminant mobility and redistribution from the contaminated environment [45]. In addition, the incorporation of organic amendments into the soil, such as, compost or manure, improves the physical properties of the soil and its ability to retain water and nutrients [46], reduces the phytotoxicity of trace elements by complexing them into insoluble compounds [40], and provides plants with slow-release nutrients, improving the overall conditions for land reclamation [46]. As shown by Henderson et al. [47], the addition of compost (20%) helped to accelerate the degradation of petroleum hydrocarbons and reduce their levels in diesel-contaminated soils by 42% compared to a control series (without compost addition). According to the authors, after two years of experimentation, the levels of F2 hydrocarbons (carbon length > C10–C16), which are the most toxic to plants, did not exceed the national guidelines for agricultural, commercial, and industrial land use. 

Petroleum hydrocarbons have a toxic effect on all elements of the ecological system, and their leakage can turn soils into technological deserts with completely inhibited biological activity [6]. Given the magnitude of the problem and the need to develop sustainable and environmentally friendly remediation strategies for sites contaminated with petroleum hydrocarbons, our study was conducted to determine the feasibility of using organic and mineral materials to reduce the impact of petrol contamination on the heavy metal content of soils. The organic material used was compost and the mineral materials used were bentonite and calcium oxide. Two research hypotheses were put forward: (1) petrol, as a petroleum derivative, has a negative effect on soils by increasing the trace element content of the soil, (2) compost, bentonite, and CaO can be effectively used in the in situ stabilisation of soils which are under pressure from petroleum substances.

## 2. Materials and Methods

### 2.1. Pot Vegetative Experiment

This research was based on a rigorous vegetation pot experiment conducted in the vegetation hall of the University of Warmia and Mazury in Olsztyn (Poland). A humus layer of Eutric Cambisol soil with a sandy loam granulometric composition [48] and the following properties was used for the experiment: pH_KCl_—5.10; content of total organic carbon (TOC)—8.54 g kg^−1^, available phosphorus—34.35 mg kg^−1^, potassium—75.26 mg kg^−1^, and magnesium—41.22 mg kg^−1^. This was a two-factor experiment with increasing doses of petrol (95 unleaded): 0; 2.5; 5; and 10 cm^3^ kg^−1^ of soil. Organic (compost) and mineral (bentonite—BDC, Niepołomice, Poland and 50% calcium oxide—Zakład Obrotu Towarami Sp. z o.o., Dwikozy, Poland) materials were used to reduce the effect of petrol on the soil. The compost was composted for a period of 6 months. Leaves (about 44%), manure (about 33%), and peat (about 23%) were used to make it. The doses of compost, bentonite, and CaO were, respectively, as follows: 30 g, 20 g, and 1.47 g kg^−1^ of soil. To meet the post-emergence needs of the test plants, the soil in each pot was amended with nitrogen—150 mg [CO(NH_2_)_2_]; phosphorus—30 mg [KH_2_PO_4_]; potassium—75 mg [KH_2_PO_4_+KCl]; magnesium—50 mg [MgSO_4_∙7H_2_O]; manganese—5 mg [MnCl_2_∙4H_2_O]; molybdenum—5 mg [(NH_4_)_6_Mo_7_O_24_∙4H_2_O]; and boron—0.33 mg kg^−1^ soil [H_3_BO_3_]. Both petrol and the above materials and elements were thoroughly mixed with the 9 kg of soil and placed in polyethylene pots. A constant soil moisture was maintained throughout the experiments. Each trial site had four replicates. The soil samples were taken for laboratory analysis after the test plant (oat) harvest at the panicle emergence stage.

The petrol doses and soil amendments used in this experiment were selected based on the results of previous preliminary studies. The predominant mechanism of the interaction of bentonite with trace elements is ion exchange (complexation in the outer sphere) and sorption (precipitation) by the binding of the metal ions in the inner sphere to the edges of the minerals [49,50]. Calcium oxide has an indirect effect on the trace element content by increasing both the soil pH and the negative charge of soil particles, thereby increasing the adsorption of the positively charged metal ions and their precipitation as hydroxides [40]. On the other hand, the immobilising effect of compost is based on the formation of complex combinations with trace elements, of the chelate type, with the highly humified organic matter [51].

### 2.2. Analytical Methods

**Figure 1:**
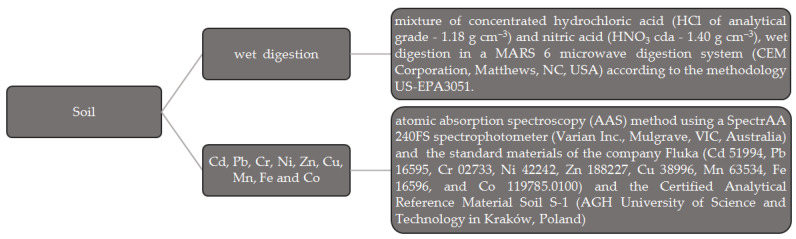
Methods of soil analysis [52,53].

After air drying and sieving, the soil samples were wet-digested for the determination of heavy metals according to the US-EPA3051 method [52]. A detailed description of the analytical methods used for heavy metals [52,53], as determined by the experimental design, is given in Figure 1.

### 2.3. Statistical Methods

For the statistical analysis of the results obtained in the study, Statistica software version 13.3 [54] was used, and the standard deviation and percentage of observed variability were calculated for the factors studied using the η^2^ coefficient from the ANOVA method, and analysis of the Anova two-factor variance and PCA (Principal Component Analysis) were performed at a significance level of *p* ≤ 0.01.

Changes in the content of trace elements in the soil as a function of petrol dose and material amendments were evaluated using a two-way analysis of variance (ANOVA) at *p* ≤ 0.01 and a post-hoc Tukey test (HDS). ANOVA is a very good statistical method used for statistical calculations of significant differences between the averages of many (three or more) groups. The PCA is used to find the relationship between the tested variables based on the designation of linear components between them. The experiments were performed in four replicates and the values are presented as the mean ± standard deviation. 

The percentage of variability (η^2^) was calculated from the formula [54]:

η^2^ = SS effect/SS Total SS × 100%

where:

η^2^—coefficient η^2^,
SS effect—the sum of squares related to a given effect,
And Total SS—the sum of squares associated with all effects.

## 3. Results

### 3.1. Heavy Metals

**Figure 2:**
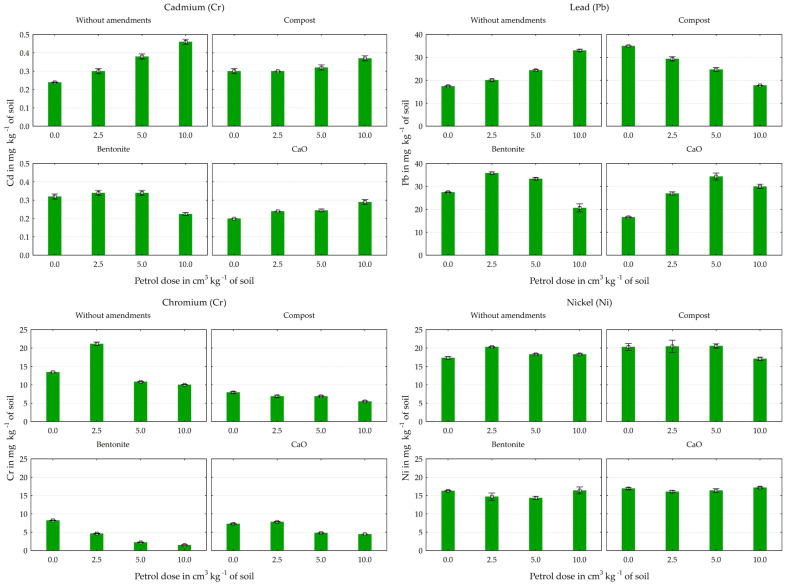
Effect of petrol and material amendments on Cd, Pb, Cr, and Ni content in soil, in mg kg^−1^ (averages ± standard deviations).

**Figure 3:**
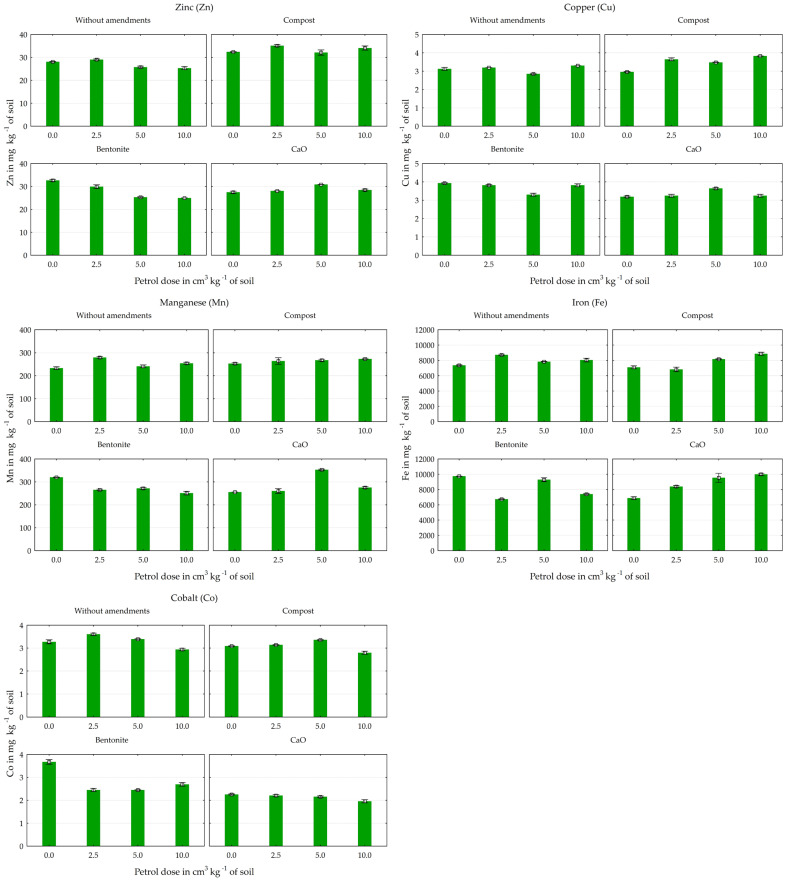
Effect of petrol and material amendments on Zn, Cu, Mn, Fe, and Co content in soil, in mg kg^−1^ (averages ± standard deviations).

Petrol contamination had a significant effect on the heavy metal content of the soil (Figure 2 and Figure 3). In the series without any material application, the Pb and Cd contents in the soil increased by 90% and 93%, respectively, as a result of this petroleum–substance interaction, compared to the control group, without petrol. The lowest dose of petrol also contributed to an increase in the content of most of the other heavy metals in the soil. Under its influence (in the unsupplemented series) there were increases of 3% in Zn, 10% in Co, 17% in Ni, 19% in Fe, 20% in Mn, and 57% in Cr. Higher doses of petrol reduced the content of these elements, especially of Co and Zn by 10% and Cr by 26%, compared to the control (without this petroleum substance). 

**Figure 4:**
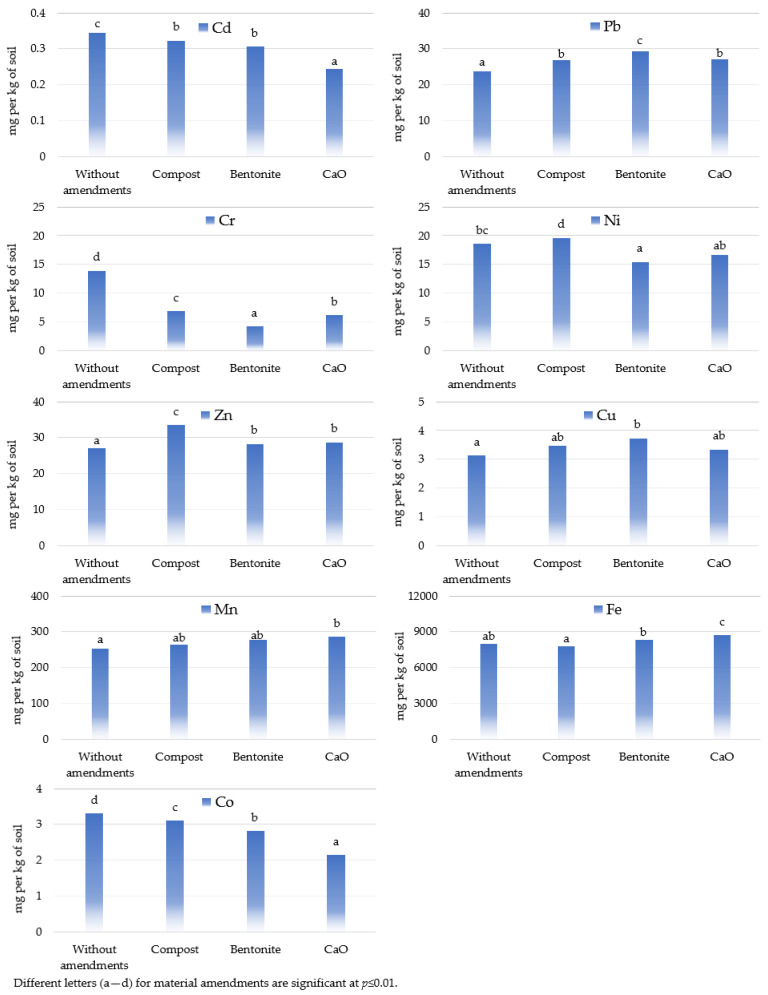
Effect of material amendments on heavy metal content in soil, in mg kg^−1^ (averages for all objects in series).

The applied mitigating substances generally had a positive (reducing) effect on the content of most of the elements tested in the soil (Figure 2, Figure 3 and Figure 4). All the substances used in the experiment resulted in a reduction in the content of Cd, Co, and especially Cr compared to the unsupplemented series. The average reductions (for all objects in series for each metal: Cd, Co, and Cr) were 7%, 6%, and 51% for compost, 11%, 15%, and 70% for bentonite, and 29%, 35%, and 56% for calcium oxide. Calcium oxide and bentonite had a similar effect on the soil’s Ni content, reducing it by 10% and 17% on average. Inverse relationships were found for the Zn, Cu, and Pb contents of the soil. Calcium oxide increased their content by an average of 6%, 7%, and 14%, compost by 24%, 12%, and 13%, and bentonite by 4%, 19%, and 23% compared to the control series (without materials). Calcium oxide had a similar but much weaker effect on the soil’s Fe content, increasing its accumulation by 9%. However, it should be noted that bentonite and compost had a limiting effect on the Pb content at the highest petrol contamination level (10 cm^3^ kg^−1^ soil). 

### 3.2. Relations between Heavy Metals

**Figure 5:**
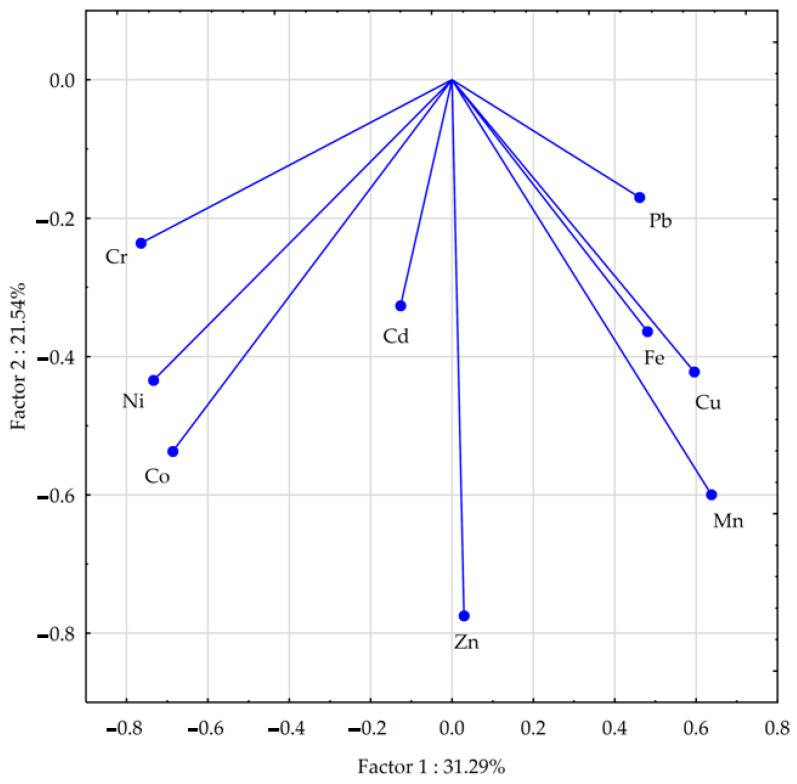
Relations between heavy metal content in soil calculated with PCA method.

**Figure 6:**
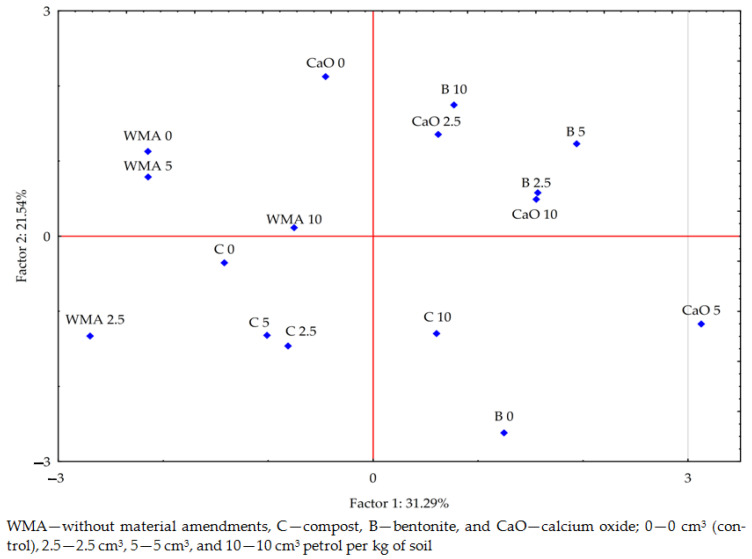
Relative effects of petrol and materials on heavy metal content in soil calculated with PCA method.

The PCA analysis carried out indicates the existence of correlations between heavy metals in the petrol-contaminated soil after the application of the materials to the soil (for all research combinations—Figure 5). The vector variables presented strong positive correlations between Cr, Ni, and Co; and between Mn and Cu, and weaker positive correlations between Mn, Fe, and Pb. Negative but relatively weak correlations were also found between Cr and Pb. The scatter of the points in Figure 6 indicates that calcium oxide and especially bentonite had a greater effect on the content of the analysed heavy metals in the soil than compost.

**Figure 7:**
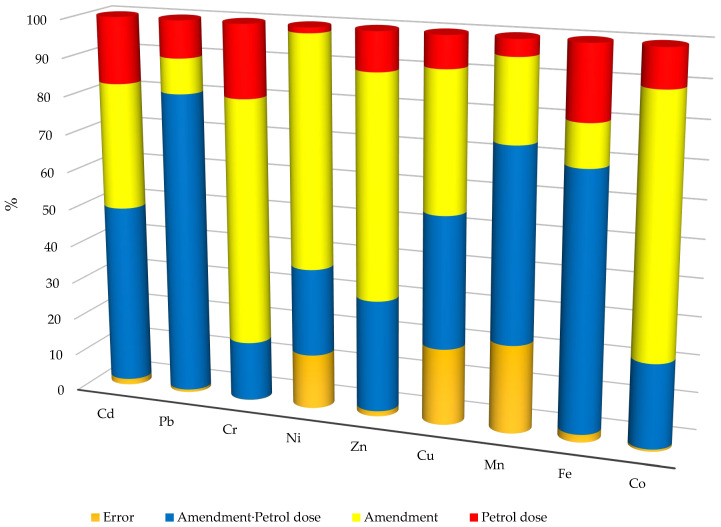
Relatively effect of factors on heavy metals content in soil (in per cent).

The calculated percentage of the observed variability using the coefficient η2 from the ANOVA method indicates that the type of neutralisation material had a greater effect than the soil contamination with petrol on the heavy metal content of the soil (Figure 7, Table S1). The effect of materials was dominant for Cu (36.65%), Zn (58.81%), Ni (60.95%), and Cr (64.99%) contents. There was also a significant effect of materials on the Cd content (33.30%) of the soil. The effect of petrol contamination on the heavy metal content of the soil was significantly lower. A high proportion of interaction of materials with soils contaminated by petrol was also observed for the variables studied. It was highest for Cd (47.68%), Mn (50.88%), and Pb (80.26%) and significant for Zn (29.65%) and Cu (34.90%).

## 4. Discussion

In our study, increasing doses of petrol caused an increase in the content of most of the trace elements analysed, with the exception of Cr, Zn, and Co. However, the most significant changes were observed for Pb and Cd, whose content in the soil increased by 90% and 93%, respectively, compared to the control (the non-contaminated group). This is in agreement with the results of our previous experiments [27]. They showed a positive correlation between soil contamination with petrol at a dose of 10 cm^3^ kg^−1^ and the content of Cd, Pb, Ni, and Cu. Compared to the control site, the content of Cd and Pb in the soil increased by almost two times, with Ni by 17% and Cu by 12%. The opposite trend was observed for Cr, Zn, and Co. An analogous effect of soil contamination by petroleum waste on the content of Cd, Cu, Ni, and Pb was shown by Sattar et al. [55]. Similar observations were also reported by Aradhi et al. [56] who investigated the trace element content of soils around oil and gas wells in the East and West Godavari districts of India. Based on the results, the authors found elevated levels of Cu, Cr, Zn, and Ni in the collected soil samples compared to a control (uncontaminated) series. A close relationship between the trace element accumulation in soils and oil exploration, production and processing activities has also been demonstrated by Qaiser et al. [57] and Ekperusi et al. [58]. 

The elevated levels of trace elements in petroleum-contaminated soils are probably a consequence of their presence in the petroleum product itself. In a study of crude oil mixtures, Dickson and Udoessien [59] identified Zn, Pb, Mn, Co, Cd, Fe, Ni, Cr, Cu, and V in them. In addition, the authors reported high levels of Ni, V, and Fe, indicating crude oil as a potential source of environmental trace element contamination in the event of a spill or pipeline failure. Petroleum fuels also affect the physicochemical properties of contaminated soils, including the sum of exchangeable base cations and total exchange capacity [2], conductivity, chloride and total nitrogen content [58], and pH [60], which is reduced [13]. This leads to an increase in the migratory mobility of trace elements and the content of their mobile forms [61]. In addition, at a low pH the content of OH^-^ groups is reduced, resulting in less attraction of trace element ions and a reduction in their precipitation as hydroxides, sludge, and mineral residues [62]. This may explain the accumulation of Pb, Cd, Cr, Co, Ni, Fe, and Mn in soils contaminated with petrol at a dose of 2.5 cm^3^ kg^−1^ found in this study.

In our study, the addition of substances that neutralise the toxic effect of petrol on the soil contributed to a reduction in the trace element content in soil. The greatest changes were observed in the series with calcium oxide, where the content of Cr (by 56%), Co (by 35%), Cd (by 29%), and Ni (by 10%) was reduced in comparison with the control (without the additive). The usefulness of calcium oxide for the immobilisation of trace elements in contaminated soils was also confirmed by Kosiorek and Wyszkowski [26]. After its application, the authors observed a reduction in Cr (by 78%), Fe (by 42%), Mn (by 41%), and Cd (by 34%) compared to the control soil. A reduction of Cd, Cr, Ni, and Co in petrol-contaminated soil after the calcium oxide application was also reported in our previous study [27]. The introduction of alkaline additives into the soil reduces the solubility of trace elements and their bioavailability in the soil due to their increased sorption to colloidal particles (e.g., clay, organic matter, and Fe and Al oxides) with an increased net negative charge under alkaline pH conditions [63]. This is confirmed by a study by Vondráčková et al. [64], which investigated the effect of CaO and dolomite on trace element immobilisation in contaminated soils. The authors showed that the liming process contributed to the reduction of Cd and Zn contents, while it remained unaffected with respect to Fe and Mn. The reduction in trace element mobility and availability under these conditions was due to the alkalising effect of CaO and the increase in soil pH. According to the authors [64], the application of calcium in its oxide form to wet soil resulted in a highly alkaline slaked lime, which significantly increased the soil pH (12.3 versus 6.5). Soil pH is one of the most important parameters determining the solubility, mobility and transformability of trace elements in soils [65]. For most trace elements, mobility and bioavailability decrease with increasing soil pH [66]. Alkaline soil amendments also have the effect of restoring the biological balance of contaminated soils and thus sequestering trace elements through increased microbial activity [67,68]. The beneficial effect of alkaline materials and compost used in the in situ stabilisation of contaminated soils on the reduction of trace elements has also been demonstrated by Madejón et al. [69] and Pardo et al. [70].

The application of mineral and organic substances increases the sorption capacity of the soil [71,72], alters the redox conditions, and improves the physicochemical properties of the contaminated soil. The application of bentonite and calcium oxide in the remediation of petrol-contaminated soil increased pH, total exchangeable bases, sorption capacity and base saturation [60]. 

In our own study, soil remediation with bentonite contributed to a significant reduction in the average content of Cd, Co, Ni, Pb, and Cr. Similar results were reported by Klik et al. [73], who used bentonite to stabilise in situ soils from steel landfills. After incorporation into the soil, the authors reported significant reductions in total Pb (by 13%), Cr (by 16%), Ni (by 20%), Cd (by 23%), Cu (by 24%), and Zn (by 32%) compared to the control series. Yu et al. [32] also showed that the addition of bentonite reduced the labile fractions of Cd (by 35.4%), Zn (by 36.6%), and Cu (by 37.1%) in the contaminated soil. Bentonite is characterised by its highly specific surface area [74] and its ability to absorb cations and organic matter, including polycyclic aromatic hydrocarbons [75]. In addition, the incorporation of bentonite improves the physical and chemical properties of the soil, including its ability to retain water and nutrients [76], helps to buffer the soil pH, increases the organic matter content [77], and improves the microbial and biochemical activity of soils [77,78]. All these changes contribute to the immobilisation of contaminants in the soil matrix [79] and have a beneficial effect on soil fertility and crop yields [80]. According to Yu et al. [32], the predominant mechanism of immobilisation of Cd, Zn, and Cu by bentonite is ion exchange, physical adsorption and partitioning for Hg, while for Cr and As it is specific adsorption and electrostatic attraction. 

The addition of a stabilised organic matter to the soil in the form of compost improves soil fertility and also reduces the mobility and availability of trace elements through the formation of chelated organometallic complexes [51]. A reduction in the bioavailability and content of mobile forms of Cu, Zn, and Pb after the application of compost (50 Mg ha^−1^) to the contaminated soil was shown by Alvarenga et al. [81]. According to the authors, the addition of compost also increased soil pH (5.8 versus 3.9), organic matter content, exchange capacity, available K and P forms, and total N content. In addition, it improved soil enzymatic activity and increased dehydrogenase and β-glucosidase activity, indicating the soil’s ecological recovery. Similar results were obtained by Khedr et al. [51], who demonstrated the usefulness of compost and vermicompost in immobilising Cr^3+^, Cd^2+^, and Pb^2+^. Remediation of contaminated soil with compost reduced the exchangeable fraction of Pb by 30%, Cr by 30–40%, and Cd by 40–70% relative to a control series (without the addition of a neutralising substance). Introducing compost and other organic substances into the soil allows the formation of highly polymerising particles that form stable organic forms with trace elements, reducing their availability and mobility in contaminated soils [51,82]. The above observations remain consistent with the results obtained in the present experiment, in which the application of compost contributed to a significant reduction in the accumulation of Pb and Cr in the soil, and less Co and Cd.

Some limitations of in situ stabilisation in the remediation of petrol-contaminated soils may be related to an excessive pH increase following application of the soil amendment and the immobilisation of important nutrients in the soil [73]. The materials used in this remediation technique (e.g., fly ash) may themselves be a source of trace elements or may increase their solubility and translocation to plant organs [42], depending on the environmental conditions. In addition, the effect of soil amendments on anionic elements (e.g., As) is highly variable and may depend on the final soil pH or the amount of material applied [83], while the effect of alkaline compounds in controlling trace element speciation may be reversed when the soil is reacidified [84].

The effectiveness of a method for immobilising contaminants in soil depends on many factors, including soil type, level of contamination and characteristics of the contamination sites. Further research is therefore needed, and a future direction seems to be to test the effectiveness of in situ stabilisation with selected materials under field conditions, as the present experiment was conducted in a controlled environment.

## 5. Conclusions

Petrol contamination and the materials used to reduce its impact (compost, bentonite and calcium oxygen) significantly influenced the heavy metal content of the soil. 

Petrol had the greatest effect on the content of lead and cadmium in the soil. Under the influence of petrol, a linear increase in the lead and cadmium content of soils was shown, by 90% and 93%, respectively. The lowest petrol dose also contributed to increases in soil zinc (3%), cobalt (10%), nickel (17%), iron (19%), manganese (20%), and chromium (57%). Higher petrol doses reduced the content of these elements, especially cobalt and zinc, which were 10 and 26% lower, respectively, than in the uncontaminated soil. 

The amendments used generally had a reducing effect on the content of most of the tested elements in the soil. Compost, bentonite, and calcium oxide all reduced cadmium, cobalt and especially chromium in the soil compared to the untreated series. Calcium oxide reduced cadmium (by 29%), cobalt (by 35%), and chromium (by 56%) in soil the most, while bentonite reduced chromium (by 70%). Compost had by far the least effect on the levels of these heavy metals, especially Cr (by 51%) in the soil. Calcium oxide and bentonite also reduced nickel in the soil (by 10% and 17%, respectively). An inverse relationship was found for zinc, copper, and lead. Their levels increased when calcium oxide, compost and especially bentonite were added to the soil. However, it should be noted that bentonite and compost had a limiting effect on the lead content in the case of the highest petrol contamination.

The application of various materials, especially calcium oxide and bentonite, can be effective in mitigating the effects of low levels of petrol contamination on soil properties (including heavy metal content) and consequently on plant growth and development. 

The use of these materials is therefore not only an effective method of soil reclamation, but also a factor in ensuring the balance of agricultural ecosystems.

The experimental results obtained not only increase our knowledge of the effects of petrol pollution on the content of trace elements, but also show the potential of using cheap and widely available materials, such as, compost, calcium oxide, or bentonite for the reclamation of such areas.

## Data Availability

Data are contained within the article.

## Supplementary Materials

The following supporting information can be downloaded at: https://www.mdpi.com/article/10.3390/ma17143528/s1, Table S1: The sum of squares related to a heavy metals in soil.
