# Supplementary material for: Mineral and Organic Materials as Factors Reducing the Effect of Petrol on Heavy Metal Content in Soil
Source: Materials (Basel). 2024 Jul 16;17(14):3528. doi: 10.3390/ma17143528 (PMC11278834; doi:10.3390/ma17143528)
Supplement: Supplementary file 1 [file materials-17-03528-s001.zip › materials-3088892-supplementary.pdf]

materials

MDPI

Supplementary

Mineral and Organic Materials as Factors Reducing the Effect of Petrol on Heavy Metal Content in Soil

Mirosław Wyszkowski * and Natalia Kordala

Department of Agricultural and Environmental Chemistry, University of Warmia and Mazury in Olsztyn, Łódzki 4 Sq., 10-727 Olsztyn, Poland; miroslaw.wyszkowski@uwm.edu.pl; natalia.kordala@uwm.edu.pl
* Correspondence: miroslaw.wyszkowski@uwm.edu.pl; https://orcid.org/0000-0001-8194-6313

Table S1. The sum of squares related to a heavy metals in soil.

Effect	Cd	Pb	Cr	Ni	Zn	Cu	Mn	Fe	Co
Amendment	0.045100	125.05	432.794	84.748	187.92	1.5242	5261	4.040158E+06	6.1350
Petrol dose	0.023670	131.95	127.471	1.995	32.40	0.3458	1972	6.940962E+06	0.9044
Amendment*Petrol dose	0.064576	1084.01	105.174	32.142	94.74	1.4473	18675	2.461556E+07	2.0193
Error	0.002102	9.53	0.500	20.169	4.45	0.8415	19773	7.389823E+05	0.0525
Total	0.135449	1350.54	665.939	139.054	319.51	4.1587	45682	3.633567E+07	9.1112
